# Supplementary material for: Robust temporal adiabatic passage with perfect frequency conversion between detuned acoustic cavities
Source: Nat Commun. 2024 Feb 17;15:1478. doi: 10.1038/s41467-024-45932-6 (PMC10874448; doi:10.1038/s41467-024-45932-6)
Supplement: Supplementary file 1 — Supplementary Information [file 41467_2024_45932_MOESM1_ESM.pdf]

Supplementary material for

Robust temporal adiabatic passage with perfect frequency conversion between detuned acoustic cavities

Zhao-Xian Chen[1,*], Yu-Gui Peng[2,*], Ze-Guo Chen[3,†], Yuan Liu[1], Peng Chen[1], Xue-Feng Zhu[2,†] and Yan-Qing Lu[1,†]

[1]National Laboratory of Solid State Microstructures, Collaborative Innovation Center of Advanced Microstructures, and College of Engineering and Applied Sciences, Nanjing University, Nanjing 210093, People's Republic of China

[2]School of Physics and Innovation Institute, Huazhong University of Science and Technology, Wuhan, Hubei 430074, People's Republic of China

[3]School of Materials Science and Intelligent Engineering, Nanjing University, Suzhou 215163, People's Republic of China

[*] These authors contributed equally to this work.

[†]Corresponding authors: G.Z. (zeguoc@nju.edu.cn); X.Z. (xfzhu@hust.edu.cn); Y.L. (yqlu@nju.edu.cn);

Contents

I. Photographs of the experimental samples 2
II. Gain-enhanced acoustic cavities 4
III. Dynamic modulations for the coupling 6
IV. Parameter selection for the Gaussian modulation 10
V. Analyzing the adiabaticity of the transient passage 13
VI. Adiabatic passage for identical cavities 15
VII. Efficiency of the TQPM for adiabatic passages 18

I. Photographs of the experimental samples

In our experiments, the cuboid cavities (see Fig. S1) are precisely machined from stainless steel, and the geometric parameters of the inner volume are $w = 5\text{cm}$ and $h = 10\text{cm}$. Here we use the cavity's first-order resonant mode, which has a dipole-like anti-symmetric mode profile along the height, as shown in the inset, and is essential for designing positive and negative couplings (details for the coupling design are given in section III). Steel detuning plates are designed with different thicknesses to tune the heights of the cavities. The top cover is made of acrylic board equipped with a microphone and three speakers to realize effective gain and coupling together with other electric components, including voltage-controlled amplifiers (VCAs), phase shifters, power amplifiers, and double-pole, double-throw (DPDT) relays.

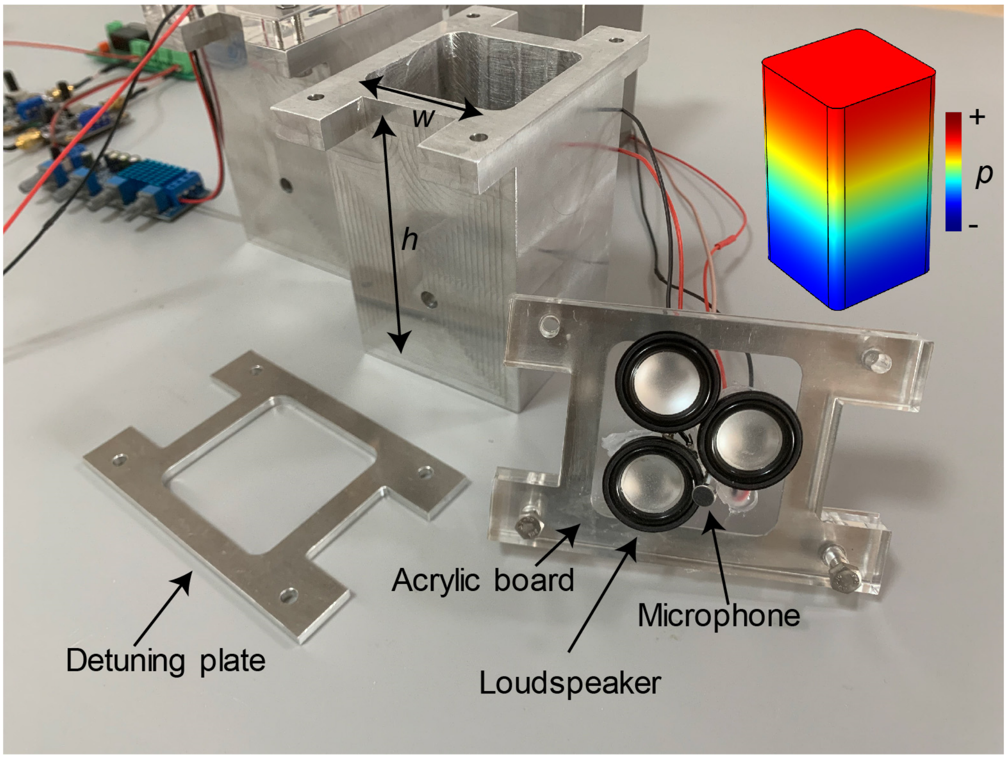

Fig. S1. A photograph of the acoustic cavity, the detuning plate, and the cover with the microphone and speakers. The inset in the top-right corner shows a dipole-like mode profile of the pressure for the first-order cavity mode simulated with finite-element methods.

Figure S2 shows the experimental setup with the dynamic coupling between two gain-enhanced cavities. For clarity, power supplies for these elements are omitted. The dynamic coupling is realized by detecting the sound in cavity **A**(**B**) with a microphone

and then feeding it to the speaker in cavity **B**(**A**) via several circuit components, including a VCA, a phase shifter, an amplifier, and a DPDT. Here, the microphone and the speakers are attached to the acrylic board and are sealed in the cavities. A lab-designed circuit is used for 100 times pre-amplification. The power amplifier model we use is TPA3116, which has two parallel channels. In the circuit for gain, only one channel of TPA3116 is used. The voltage-controlled amplifier model is VCA821 from Texas Instruments. It controls the amplification with a gate voltage. The phase shifter (model MCP41010) is introduced to compensate for the phase variation induced by the VCA. Because the VCAs cannot drive the speakers directly, the power amplifier TPA3116 is utilized in the coupling circuit. The DPDT relay is model HK-19F. The following two sections give details about the modulations of the VCA and DPDT.

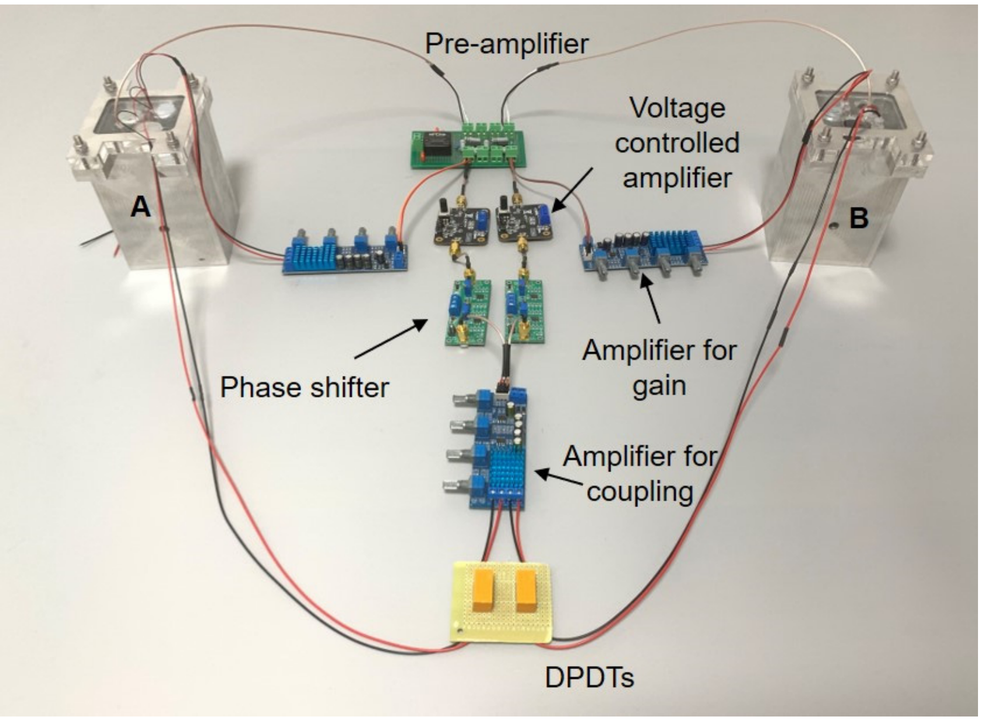

Fig. S2. Photograph of the experimental setup for the dynamic coupling between two gain-enhanced cavities. The simplified schematic is shown in Fig. 2**a** in the main text.

II. Gain-enhanced acoustic cavities

To realize the sound wave transfer, we extend the lifetime of the modes by introducing gain with an in-phase feedback circuit to compensate for the intrinsic loss of the cavities. As shown in Fig. S3**a**, both the microphone and the loudspeaker are fixed at the same side of the cavity, and the feedback circuit contains a phase shifter and a VCA, which are also used in the coupling circuit. The VCA can digitally and precisely control the amplitude of the gain. The phase shifter is used to ensure exactly in-phase gain. By digitally controlling the circuit, the measured (circles) and fitted (curves) exciting spectra in Fig. S3**b** show that the cavity's damping rate can decrease from the original $\Gamma = 10$ Hz (black) to 0.8 Hz (blue) or even 0.1 Hz (red). Thus, the acoustic mode's lifetime is extended. The damping rates can also be verified by fitting the transient damping pressures, as presented in Fig. S3**c**. Here, the sound energy is introduced by exciting the source speaker for $t \in [-0.1, 0]$ second. By using the tuning plate to change the cavity heights, the three cavities are modified to resonant at $f_{\mathrm{A}} = 1605$ Hz, $f_{\mathrm{B}} = 1655$ Hz and $f_{\mathrm{C}} = 1585$ Hz, respectively. The damping rates are calibrated at $\Gamma = 8$ Hz, which is low enough for observing the mode evolutions. The excitation spectra of the three cavities are measured and plotted in Fig. S3**d**.

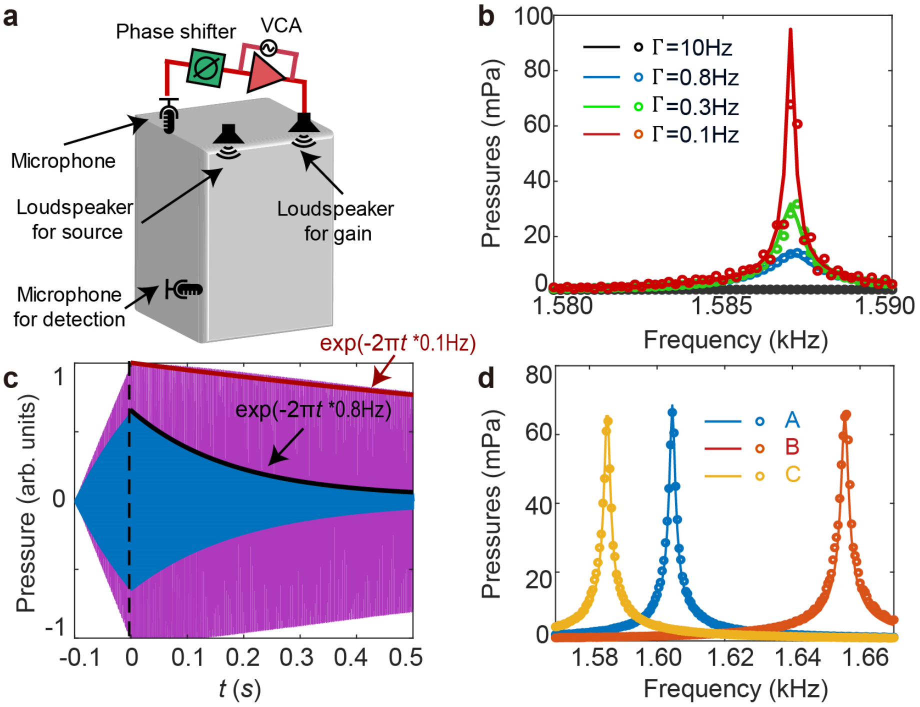

Fig. S3. Gain-enhanced acoustic cavities. **a**, A schematic view of the experimental setup that introduces exact pure gain by adopting the phase shifter and the VCA into the feedback circuit. **b**, The measured (circles) and fitted (curves) excitation spectra of a

cavity with different gain strengths. The damping rate decreases from the original $\Gamma = 10\ \text{Hz}$ (black) to $0.8\ \text{Hz}$ (blue) or even $0.1\ \text{Hz}$ (red). **c**, The transient sound wave excitation ($t < 0$) and damping ($t > 0$) in the cavity. The solid curves represent the fitted pressure amplitude variations with $\Gamma = 0.8\ \text{Hz}$ (black) and $0.1\ \text{Hz}$ (red), respectively. **d**, Excitation spectra for three gain-enhanced cavities, showing that they are resonating at frequencies $f_\text{A} = 1605\ \text{Hz}$, $f_\text{B} = 1655\ \text{Hz}$, and $f_\text{C} = 1585\ \text{Hz}$, respectively. The damping rates are calibrated at $\Gamma = 0.8\ \text{Hz}$.

III. Dynamic modulations for the coupling

The two-cavity system with a constant coupling κ_0 is represented by the Hamiltonian

$$\mathbf{H} = 2\pi \begin{bmatrix} f_\mathrm{A} - i\Gamma & \kappa_0 \\ \kappa_0 & f_\mathrm{B} - i\Gamma \end{bmatrix}, \quad \text{(S1)}$$

where f_A and f_B are the resonant frequencies of the two cavities, Γ is the damping factor. We obtain the spectral response of the two cavities by solving the wave equation

$$i\frac{d}{dt}|\psi(t)\rangle = \mathbf{H}|\psi(t)\rangle + |\mathrm{s}(t)\rangle. \quad \text{(S2)}$$

Here $|\psi(t)\rangle = \begin{bmatrix} p_\mathrm{A} \\ p_\mathrm{B} \end{bmatrix} \mathrm{e}^{-i2\pi f t}$ is the state function and $|\mathrm{s}(t)\rangle$ represents the sound source. As an example, when cavity **A** is excited, the spectral response of the two cavities is given by

$$p_\mathrm{A}(f) = \frac{(f - f_\mathrm{B} + i\Gamma)p_\mathrm{in}}{(f - f_\mathrm{A} + i\Gamma)(f - f_\mathrm{B} + i\Gamma) - \kappa_0^2}, \quad \text{(S3-1)}$$

$$p_\mathrm{B}(f) = \frac{\kappa_0 p_\mathrm{in}}{(f - f_\mathrm{A} + i\Gamma)(f - f_\mathrm{B} + i\Gamma) - \kappa_0^2}, \quad \text{(S3-2)}$$

with p_in being the amplitude of the sound source. The phase relation between the two cavities is

$$\mathrm{Arg}\left(\frac{p_\mathrm{B}(f)}{p_\mathrm{A}(f)}\right) = \mathrm{Arg}\left(\frac{\kappa_0}{f - f_\mathrm{B} + i\Gamma}\right). \quad \text{(S4)}$$

Thus, by fitting Eqs. (S3-S4) to the measured pressures and phases, we can determine the mutual coupling κ_0.

We first consider the general case (see Fig. 2 of the main text) with two detuned cavities ($f_\mathrm{A} = 1605$ Hz, $f_\mathrm{B} = 1655$ Hz, and $\Gamma = 0.8$ Hz). For example, gate voltages for the VCAs are set to 0.5V, and the phase shifters and power amplifiers are tuned to realize real-valued mutual couplings between the two cavities. The measured and fitted pressures are given in Figs. S4(**a-b**), with the fitting coupling 9.5Hz, when either cavity **A** or **B** is excited. Notably, here the coupling is positive, as seen from the phase difference given in the insets.

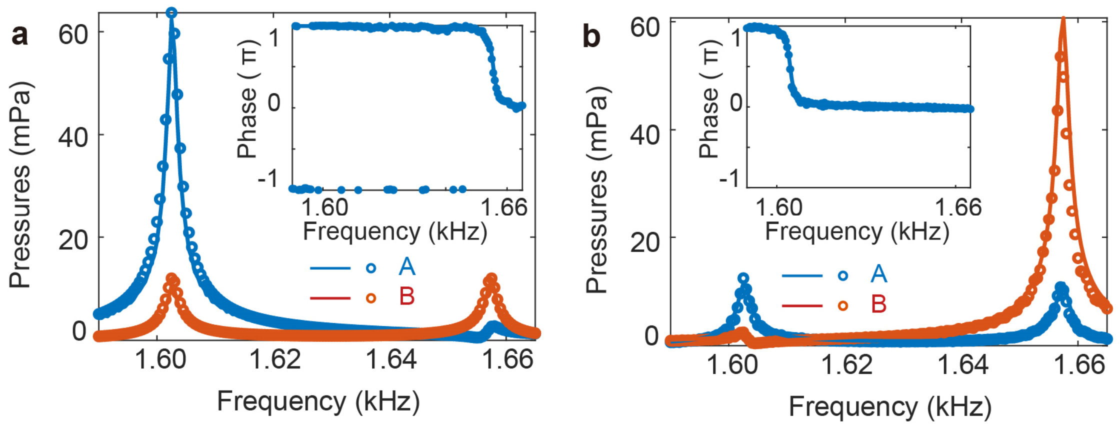

Fig. S4. Measured (circles) and fitted pressures (curves) of the two cavities with **A** (**a**) or **B** (**b**) being excited. The resonance frequencies are $f_{\mathrm{A}} = 1605$ Hz, $f_{\mathrm{B}} = 1655$ Hz, and the damping is $\Gamma = 0.8$ Hz. The strength of the mutual coupling is $\kappa_0 = 9.5$ Hz. The coupling is positive (i.e., in-phase) according to the relative phase in the insets.

By using external voltage signals to control the DPDT relays, we switch the connection of the coupling circuit from in-phase to out-of-phase, which is schematically shown in Fig. S5**a**. Accordingly, the effective coupling changes from positive to negative values. Figure S5**b** gives the measured and fitted phase variation for in-phase to out-of-phase coupling.

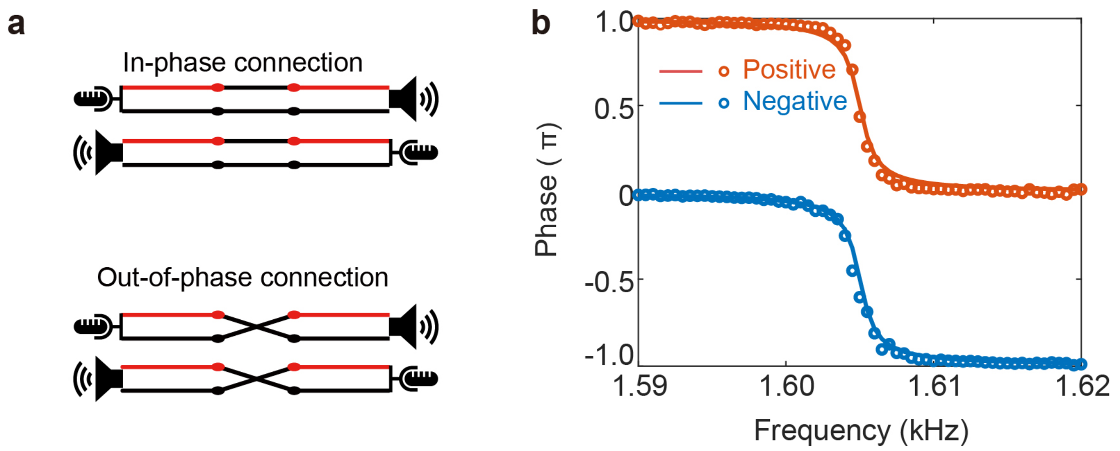

Fig. S5. **a**, The effective coupling varies from positive to negative due to switching the coupling circuit from in-phase (upper panel) to out-of-phase (lower panel) connections. **b**, The measured (circles) and fitted (curves) phase difference between two cavities for the positive (brown) or negative (blue) coupling.

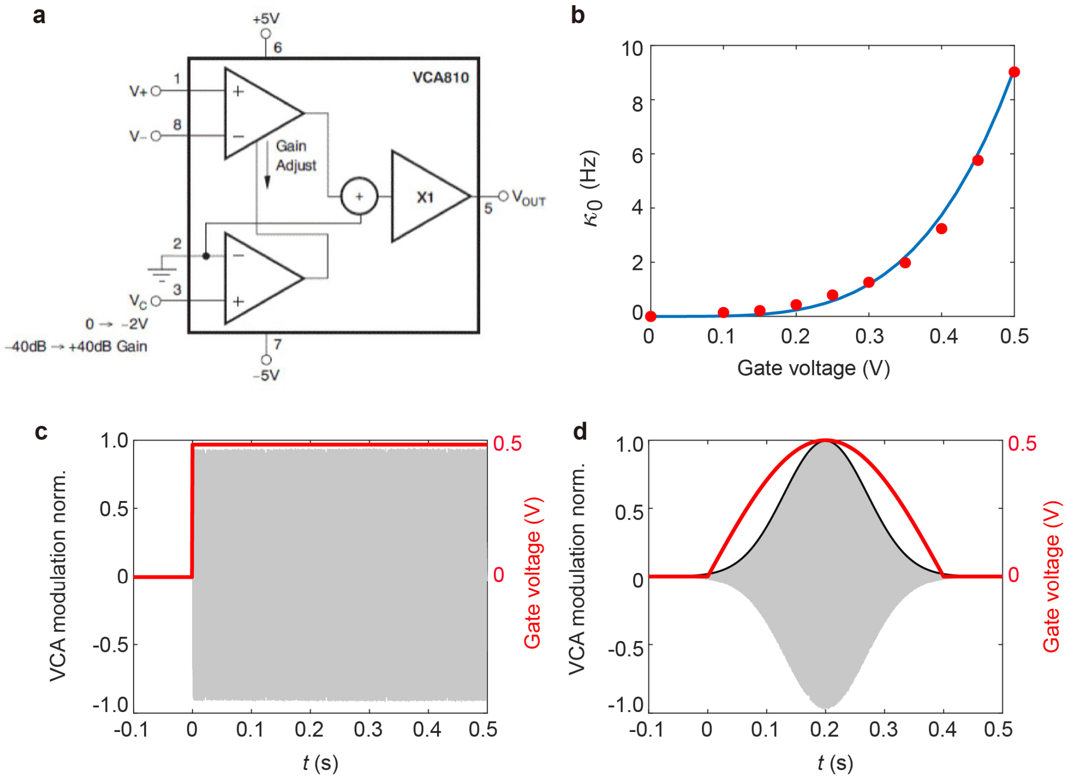

Fig. S6. **a**, Simplified electric circuit of the voltage-controlled amplifier Module 810. **b**, Variation of the measured (dots) and fitted (curve) coupling strength κ_0 with the gate voltage. **c-d**, Measured VCA modulation for step function (**c**) and Gaussian function (**d**). A sinusoidal signal is applied to the VCA input, and the red curves are the applied modulating voltage signals. The black curve in **d** is the fitted Gaussian function $e^{-(t-t_0)^2/\sigma^2}$ with $t_0 = 0.2$ s and $\sigma = 0.1$ s.

Another specific feature of our system is that the variation of the strength of the effective coupling can be programmed. To achieve this, VCAs (with the core circuit shown in Fig. S6**a**) are introduced into the coupling circuit. The coupling strength is proportional to the VCA amplifications and can be controlled with a gate voltage signal. We measure the coupling strength κ_0 as a function of the gate voltage, and the one-to-one correspondence presented in Fig. S6**b** verifies its tunability. To test the transient response of VCAs, we use a sinusoidal signal $\sin(2\pi ft)$ as the input and apply a step voltage $V(t > 0) = 0.5$ V as the control signal. The recorded transient output of the VCA in Fig. S6**c** demonstrates the immediate response between the gate voltage signal and the VCA modulation. As shown in Fig. S6**d**, to realize the Gaussian-type output, we use a half-period sinusoidal voltage (the red curve) as the control signal to tailor the VCA modulation. The resulting VCA modulation can be fitted by $e^{-(t-t_0)^2/\sigma^2}$ with $t_0 =$

$0.2\ \mathrm{s}$ and $\sigma = 0.1\ \mathrm{s}$ (the black curve). Thus, the coupling strength is also modulated as a Gaussian function because it is proportional to the amplifications of the VCAs.

IV. Parameter selection for the Gaussian modulation

For the three-cavity system with two Gaussian-type dynamic couplings, the energy transfer is determined by two parameters $\theta(t) = \arctan[\tilde{\kappa}_{AB}(t)/\tilde{\kappa}_{BC}(t)]$ and $\alpha(t) = \int_0^t \mathrm{real}[\varepsilon_+(t')]dt'$, where $\tilde{\kappa}_{AB}(t)$ and $\tilde{\kappa}_{BC}(t)$ are the effective coupling strengths, $\varepsilon_+(t) = -i\Gamma + \sqrt{\tilde{\kappa}_{AB}^2(t) + \tilde{\kappa}_{BC}^2(t)}$ is the positive eigenvalue of the effective Hamiltonian in Eq. (7) in the main text.

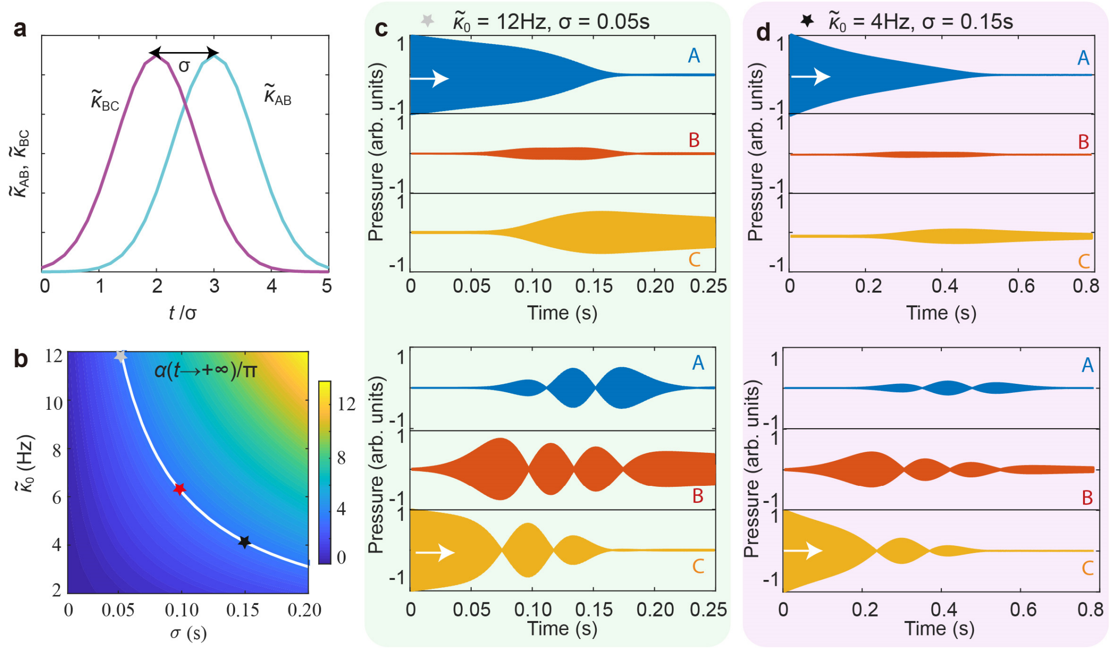

Fig. S7. **a**, Dynamic couplings $\tilde{\kappa}_{AB}(t)$ and $\tilde{\kappa}_{BC}(t)$ for the three-cavity system. **b**, Calculated $\alpha(t \to +\infty)$ as a function of $\tilde{\kappa}_0$ and σ. The white curve denotes the case of $\alpha(t \to +\infty) = 3.5\pi$, and the red star indicates the case with $\tilde{\kappa}_0 = 6.1$ Hz and $\sigma = 0.1$ s, for which results are shown in Figs. 3 and 4 of the main text. The gray and black stars denote the cases simulated in **c-d**. **c-d**, Simulated wave evolutions from **A** to **C** (upper panel) and **C** to **B** (lower panel) with (**c**) $\tilde{\kappa}_0 = 12$ Hz and $\sigma = 0.05$ s and (**d**) $\tilde{\kappa}_0 = 4$ Hz and $\sigma = 0.15$ s. The damping rates for the cavities are $\Gamma = 0.8$ Hz.

Without loss of generality, here we set $t_0 = 2\sigma$ and $\Delta t = \sigma$ in $\tilde{\kappa}_{AB}(t) = \tilde{\kappa}_0 e^{-(t-t_0-\Delta t)^2/\sigma^2}$ and $\tilde{\kappa}_{BC}(t) = \tilde{\kappa}_0 e^{-(t-t_0)^2/\sigma^2}$, so that the dynamic couplings are non-zero from 0 to 5σ, see Fig. S7**a**. We perform the integration and get $\alpha(t \to +\infty)$ as a function of $\tilde{\kappa}_0$ and σ. As shown in Fig. S7**b**, larger $\tilde{\kappa}_0$ and σ contribute to larger $\alpha(t \to +\infty)$, which improves the adiabaticity of the state evolutions (see section V for the details). For the case with $\alpha(t \to +\infty) = 3.5\pi$, as denoted by the white curve, there

is an inversely proportional relationship between $\tilde{\kappa}_0$ and σ. We simulate the energy transfers for both the forward and backward directions for $\tilde{\kappa}_0 = 12$ Hz and $\sigma = 0.05$ s ($\tilde{\kappa}_0 = 4$ Hz and $\sigma = 0.15$ s). The results are presented in Figs. 7**c**-7**d**. Obviously, larger σ needs smaller coupling strength $\tilde{\kappa}_0$ to realize the adiabaticity, but the longer modulation time (5σ) leads to smaller residual energy compared with the initial state at $t = 0$ s. By contrast, a smaller σ means shorter modulation time but requires a larger $\tilde{\kappa}_0$. Considering both the feasibility of the coupling strength $\tilde{\kappa}_0$ and the damping rates of cavities, we make a compromise and choose $\tilde{\kappa}_0 = 6.1$ Hz and $\sigma = 0.1$ s for the experiment, as denoted by the red star in Fig. 7**b**.

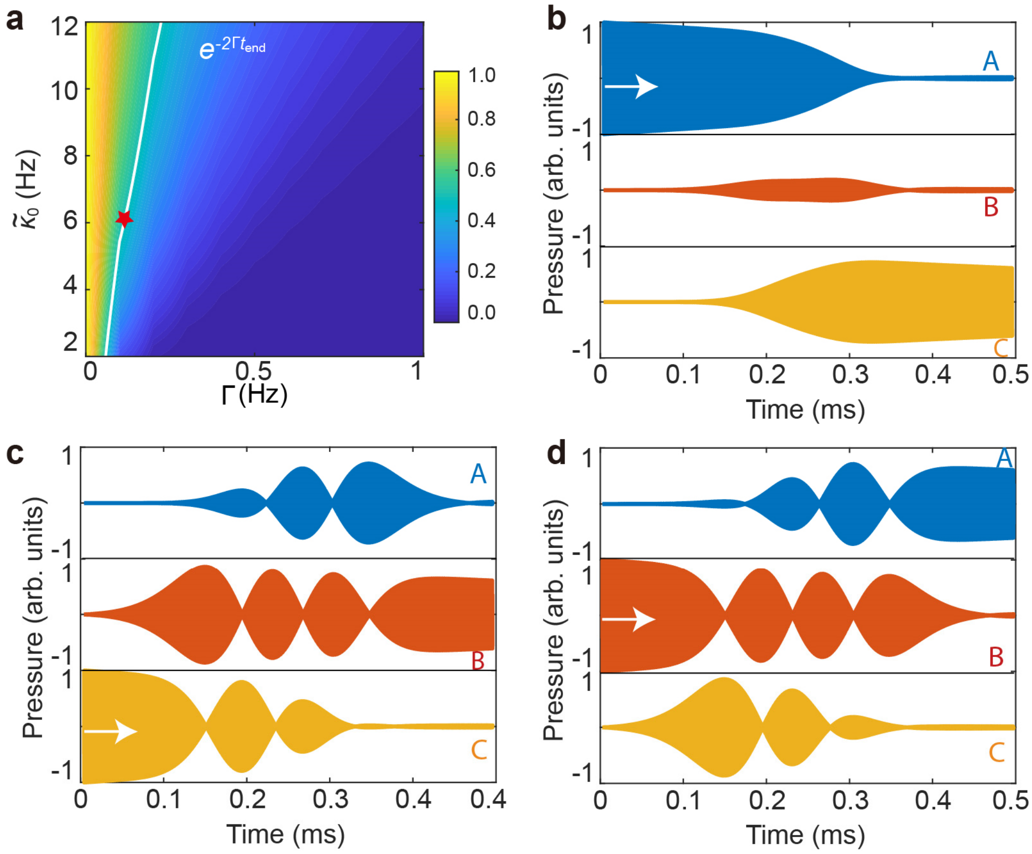

Fig. S8 **a**, Calculated values of $e^{-2\Gamma t_{\text{end}}}$ as a function of $\tilde{\kappa}_0$ and Γ. The white line indicates the contour of 0.5, and the red star denotes the parameters of $\tilde{\kappa}_0 = 6.1$ Hz and $\Gamma = 0.11$ Hz used in **b-d**. **b-d**, Simulated sound wave transfer from cavities **A** to **C** (**b**), **C** to **B** (**c**) and **B** to **A** (**d**).

When compared with the input energy at $t = 0$, the **total transfer efficiency** is defined as $|\widetilde{S_{\text{CA}}}|^2 = |p_{\text{C}}(t_{\text{end}})|^2 / \sum_j |p_j(t=0)|^2 = |S_{\text{CA}}|^2 e^{-2\Gamma t_{\text{end}}}$ with $t_{\text{end}} = t_0 +$

$2\sigma + \Delta t$ being the time point after the modulation. Apparently, $|\widetilde{S_{\mathrm{CA}}}|^2$ can be effectively improved twofold, *i.e.*, decreasing the cavity damping Γ by introducing more gain and shortening the modulation time t_{end} by enlarging coupling amplitudes. For the case with $\alpha(+\infty) = 3.5\pi$, we simulate $e^{-2\Gamma t_{\mathrm{end}}}$ as a function of $\tilde{\kappa}_0$ and Γ. Notably, according to Fig. S7**b**, here t_{end} is determined by $\tilde{\kappa}_0$ when $\alpha(+\infty)$ is fixed. Consistent with the theoretical prediction, Fig. S8**a** shows that $e^{-2\Gamma t_{\mathrm{end}}}$ can be effectively enlarged by decreasing the damping rate Γ and increasing the coupling strength $\tilde{\kappa}_0$. Figures S8**b-d** show the simulated wave transfer with $\tilde{\kappa}_0 = 6.1$ Hz and $\Gamma = 0.11$ Hz (the red star in Fig. S8a), with which the **total transfer efficiency** can be up to 0.5. The experimental results in Supplementary section II show that, by using more advanced amplifiers (such as the voltage-controlled amplifiers in the coupling circuit) to introduce gain more precisely, the cavity damping can be exactly optimized to a much lower level.

V. Analyzing the adiabaticity of the transient passage

As discussed in the main text, the robust transfer channel in our three-cavity system is determined by the adiabatic evolution of eigenstates. Under the adiabatic condition, mutual couplings between eigenstates are always negligible. Here we investigate the adiabaticity of the transient acoustic passages by analyzing the state function with the eigenvectors $\{|\psi_+(t)\rangle, |\psi_0(t)\rangle, |\psi_-(t)\rangle\}$ rather than with eigenfunction for individual cavities $\{|\varphi_A(t)\rangle, |\varphi_B(t)\rangle, |\varphi_C(t)\rangle\}$. Through the unitary transformation, the transient state function $\psi(t)$ in the new basis vectors obeys the transformed Schrödinger-type equation [1, 2]

$$i\frac{d}{dt}|\psi(t)\rangle = [\mathbf{U}^\dagger \mathbf{H}_{\mathrm{TQPM}}(t)\mathbf{U} - i\mathbf{U}^\dagger \frac{\partial}{\partial t}\mathbf{U}]|\psi(t)\rangle, \tag{S5}$$

where the unitary transformation matrix $\mathbf{U}$ is

$$\mathbf{U} = \frac{1}{\sqrt{2}}\begin{bmatrix} \sin\theta(t) & \sqrt{2}\cos\theta(t) & \sin\theta(t) \\ 1 & 0 & -1 \\ \cos\theta(t) & -\sqrt{2}\sin\theta(t) & \cos\theta(t) \end{bmatrix}. \tag{S6}$$

The parameter $\theta(t)$ is defined as $\theta(t) = \arctan[\tilde{\kappa}_{\mathrm{AB}}(t)/\tilde{\kappa}_{\mathrm{BC}}(t)]$, where $\tilde{\kappa}_{\mathrm{AB}}(t)$ and $\tilde{\kappa}_{\mathrm{BC}}(t)$ are Gaussian-shaped dynamic couplings between the cavities. For the evolution of the three-cavity system with dynamic couplings, we recall the Hamiltonian with the temporal quasi-phase matching as

$$\mathbf{H}_{\mathrm{TQPM}}(t) = 2\pi\begin{bmatrix} -i\Gamma & \tilde{\kappa}_{\mathrm{AB}}(t) & 0 \\ \tilde{\kappa}_{\mathrm{AB}}(t) & -i\Gamma & \tilde{\kappa}_{\mathrm{BC}}(t) \\ 0 & \tilde{\kappa}_{\mathrm{BC}}(t) & -i\Gamma \end{bmatrix}. \tag{S7}$$

By substituting Eqs. (S7) and (S6) into Eq. (S5), we find the systematic Hamiltonian $\mathbf{D}(t)$ with the eigenstate vectors $\{|\psi_+(t)\rangle, |\psi_0(t)\rangle, |\psi_-(t)\rangle\}$ as

$$\begin{aligned} \mathbf{D}(t) &= \mathbf{U}^\dagger \mathbf{H}_{\mathrm{TQPM}}(t)\mathbf{U} - i\mathbf{U}^\dagger \frac{\partial}{\partial t}\mathbf{U} \\ &= \begin{bmatrix} \varepsilon_+(t) & i\dot{\theta}(t)/\sqrt{2} & 0 \\ -i\dot{\theta}(t)/\sqrt{2} & 0 & -i\dot{\theta}(t)/\sqrt{2} \\ 0 & i\dot{\theta}(t)/\sqrt{2} & \varepsilon_-(t) \end{bmatrix}. \end{aligned} \tag{S8}$$

We note that the diagonal elements are the eigenvalues and the off-diagonal elements are the couplings between the zero-energy eigenstate $|\psi_0(t)\rangle$ and the other two states $|\psi_\pm(t)\rangle$. In addition, there is no direct coupling between the eigenstates $|\psi_+(t)\rangle$ and $|\psi_-(t)\rangle$. To guarantee adiabatic evolution, the rate of change of the mixing angle $\dot{\theta}(t)$ must be much smaller than the difference of the eigenvalues

$$\left|\langle\dot{\psi}_0(t)|\psi_\pm(t)\rangle\right| = \frac{1}{\sqrt{2}}|\dot{\theta}(t)| \ll |\varepsilon_0(t) - \varepsilon_\pm(t)| = \mathrm{Real}(\varepsilon_+(t)) \tag{S9}$$

with

$$\dot{\theta}(t) = \frac{\dot{\tilde{\kappa}}_{\mathrm{AB}}(t)\tilde{\kappa}_{\mathrm{BC}}(t) - \tilde{\kappa}_{\mathrm{AB}}(t)\dot{\tilde{\kappa}}_{\mathrm{BC}}(t)}{\tilde{\kappa}_{\mathrm{AB}}^2(t) + \tilde{\kappa}_{\mathrm{BC}}^2(t)}. \tag{S10}$$

This is the "local" adiabaticity condition, which should be satisfied at any time. Since there are derivatives in the numerator, the couplings $\tilde{\kappa}_{\mathrm{AB}}(t)$ and $\tilde{\kappa}_{\mathrm{BC}}(t)$ should vary smoothly for satisfying the adiabatic condition. We can also analyze the adiabatic condition by integrating Eq. (S9)

$$\int_0^\infty |\dot{\theta}(t)|\, dt \ll \sqrt{2}\int_0^\infty \mathrm{Real}(\varepsilon_+(t))\, dt. \tag{S11}$$

With $\tilde{\kappa}_{\mathrm{AB}}(t) = \tilde{\kappa}_0 e^{-(t-t_0-\Delta t)^2/\sigma^2}$ and $\tilde{\kappa}_{\mathrm{BC}}(t) = \tilde{\kappa}_0 e^{-(t-t_0)^2/\sigma^2}$ (σ is fitted as 0.1 s, $\tilde{\kappa}_0 = 6.1$ Hz). With the time lag of the two couplings set to $\Delta t = 90$ ms, we obtain from Eq. (S11)

$$\int_0^\infty |\dot{\theta}(t)|\, dt \approx \pi/2, \tag{S12-a}$$

$$\sqrt{2}\int_0^\infty \mathrm{Real}(\varepsilon_+(t))\, dt \approx 7\sqrt{2}\pi/2, \tag{S12-b}$$

which is the "global" adiabaticity condition [*3, 4*]. Comparing Eqs. (S12-a) and (S12-b), we conclude the adiabatic limit is approximately satisfied in our three-cavity system.

VI. Adiabatic passage for identical cavities

In the main text, we focus on the case where the cavities have different resonant frequencies, and the coupling's sign varies periodically to compensate for the temporal phase mismatching between the cavities. Here, we realize the transient acoustic passages with identical cavities, where the cavities have the same resonant frequencies, indicating no phase mismatching issue. In this case, only the amplitudes of the couplings are varied.

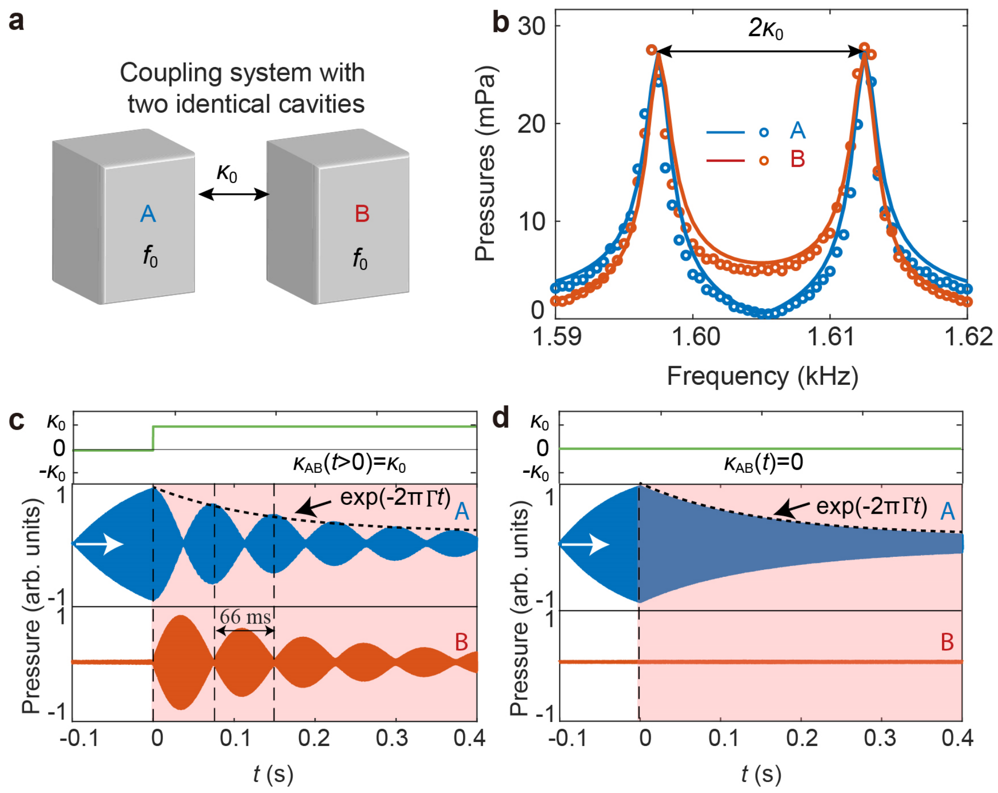

Fig. S9. **a**, Schematic of the coupling system containing two identical cavities with $f_0 = 1605$ Hz and $\Gamma = 0.8$ Hz. **b**, Spectral response of the coupling system with A being excited. The coupling amplitude can be determined from the spectral splitting as $\kappa_0 = 7.6$ Hz. **c-d**, Recorded sound waves in cavities **A** (blue) and **B** (orange), with the cavity **A** being excited for $t < 0$. The coupling (cyan line) is switched to $\kappa_0 = 7.6$ Hz in **c** after $t = 0$ and is kept zero in **d**. The black dashed lines denote the fitted damping rate with $\Gamma = 0.8$ Hz, demonstrating the coupling does not introduce an extra energy to the system.

Firstly, as shown in Fig. S9**a**, we consider a two-cavity system, where the cavities

are calibrated to resonate at $f_0 = 1605$ Hz by tuning the height of the cavities. The cavities' loss is reduced to $\Gamma = 0.8$ Hz by adjusting the in-phase feedback circuit. The measured and fitted spectra with cavity **A** being excited are shown in Fig. S9**b**. According to Eq. (S3), the coupling strength can be directly read from the splitting of the spectra, which is $2\kappa_0 = 15.2$ Hz. When cavity **A** is initially excited ($t < 0$), as shown in Fig. S9**c**, we observe complete oscillation between the two cavities after we switch on the constant coupling. The wave oscillation period is 66ms, consistent with the theoretical prediction of $0.5/\kappa_0$. In addition, by comparing with Fig. S9**d**, where the coupling is off, the wave decay in Fig. S9**c** still leads to $\Gamma = 0.8$ Hz, demonstrating that the feedback circuit for the coupling only works to transfer the sound energy between the two cavities, and does not introduce extra power to the system. In other words, the effective coupling in our system is Hermitian.

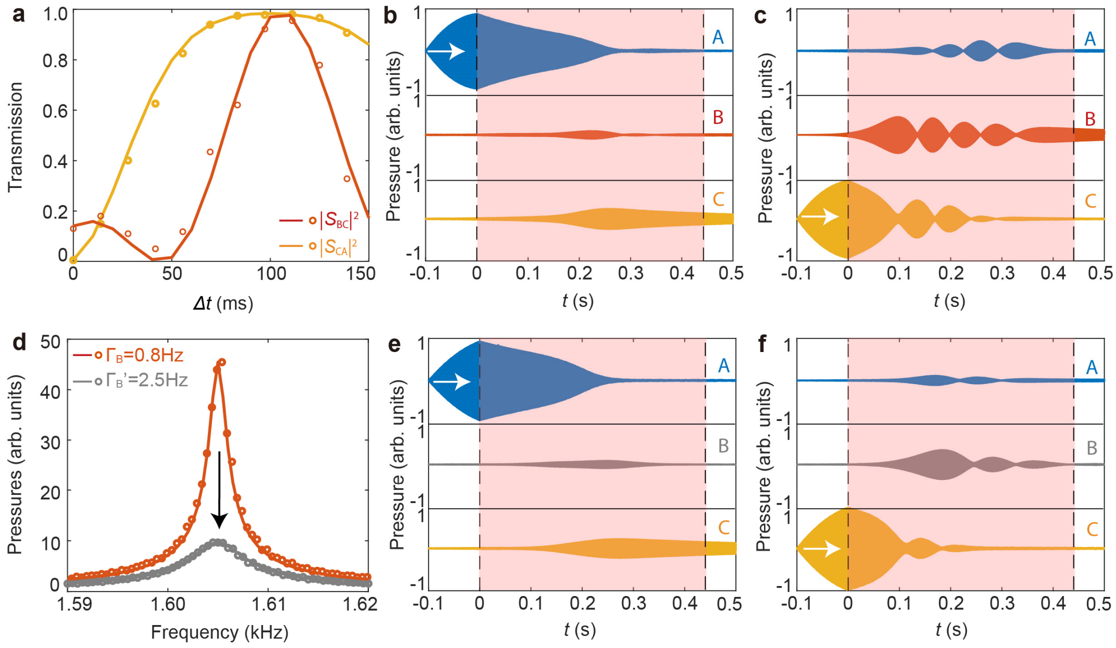

Fig. S10. **a**, Measured (circles) and simulated (curves) transfer efficiencies $|S_{CA}|^2$ and $|S_{BC}|^2$ as a function of the time delay Δt. The system consists of three identical cavities with $f_0 = 1605$ Hz and $\Gamma = 0.8$ Hz. Dynamic couplings are modulated as $\kappa_{AB}(t) = \kappa_0 e^{-(t-t_0-\Delta t)^2/\sigma^2}$ and $\kappa_{BC}(t) = \kappa_0 e^{-(t-t_0)^2/\sigma^2}$, respectively, as shown in Fig. S7**a**. **b**-**c**, Recorded sound waves in the cavities with sound excitations (white arrows) at the cavity **A** (**b**) and **C** (**c**) for $t < 0$, respectively. We set $\Delta t = 110$ ms. **d**, Measured (circles) and fitted (curves) excitation spectra for the cavity **B** with the damping rate tuned from $\Gamma_B = 0.8$ Hz (red) to $\Gamma_B' = 2.5$ Hz (gray). **e-f** are the same as **b-c** but with the cavity **B**'s damping rate set to be $\Gamma_B' = 2.5$ Hz, demonstrating that the system works

as a unidirectional absorber.

Secondly, we realize adiabatic passage by adding another identical cavity **C**. The coupling between cavities **A** and **B** (**B** and **C**) is modulated as $\kappa_{AB}(t) = \kappa_0 e^{-(t-t_0-\Delta t)^2/\sigma^2}$ ($\kappa_{BC}(t) = \kappa_0 e^{-(t-t_0)^2/\sigma^2}$), meaning that the maximum of $\kappa_{AB}(t)$ precedes $\kappa_{BC}(t)$ by Δt. Other parameters ($t_0 = 0.185$ s and $\sigma = 0.1$ s) are the same as for the Gaussian modulations discussed in the main text. The transfer among the cavities is best described by looking at the evolution of the eigenstates of the coupled system. By varying Δt from 0 to 150 ms, we measure the forward (backward) transfer efficiencies $|S_{CA}|^2$ ($|S_{BC}|^2$), given in Fig. S10**a**. The results match well the theoretical predictions obtained by solving the coupling equations. The forward transfer efficiency $|S_{CA}|^2$ approaches unity within a relatively large Δt range, but the backward passage is totally blocked because $|S_{BC}|^2$ approaches unity. For the case of $\Delta t = 110$ ms, the evolutions of the sound waves with initial excitation in cavity **A** or **C** are shown in Figs. S10**b** and S10**c**, respectively, demonstrating nonreciprocal energy transfer.

In the following, we use the method described in section V to quantitatively analyze the adiabaticity of the state evolutions as shown in Figs. S10**b-c** with $\Delta t = 110$ ms. By performing integration of the time derivative of the mixing angle and the corresponding eigenvalue, we obtain

$$\int_0^\infty |\dot{\theta}(t)|\, dt \approx \pi/2, \tag{S13-a}$$

$$\sqrt{2}\int_0^\infty \mathrm{Real}(\varepsilon_+(t))\, dt \approx 9\sqrt{2}\pi/2. \tag{S13-b}$$

Comparison of Eqs. (S13-**a**) and (S13-**b**) shows that the adiabatic condition is well satisfied.

Our system becomes a unidirectional sound absorber when extra loss is introduced to the intermediate cavity B. Figure S10**d** presents the measured excitation spectra of cavity **B** when the cavity loss increases from $\Gamma_B = 0.8$ Hz to $\Gamma_B' = 2.5$ Hz. As shown in Figs. S10**e-f**, the forward transfer from cavities **A** to **C** is well preserved, but the sound wave is almost completely dissipated in cavity **B** when the initial excitation is at cavity **C**.

VII. Efficiency of the TQPM for adiabatic passages

By proposing the TQPM method in that the coupling's sign flips at the beat frequency between the cavity modes, we overcome the temporal phase mismatching between detuning cavities and realize robust energy transfer and frequency conversion via the STIRAP process. In this section, by solving the coupled wave equations, we show that the TQPM is quite general and is effective as long as the detuning is larger than the coupling strength.

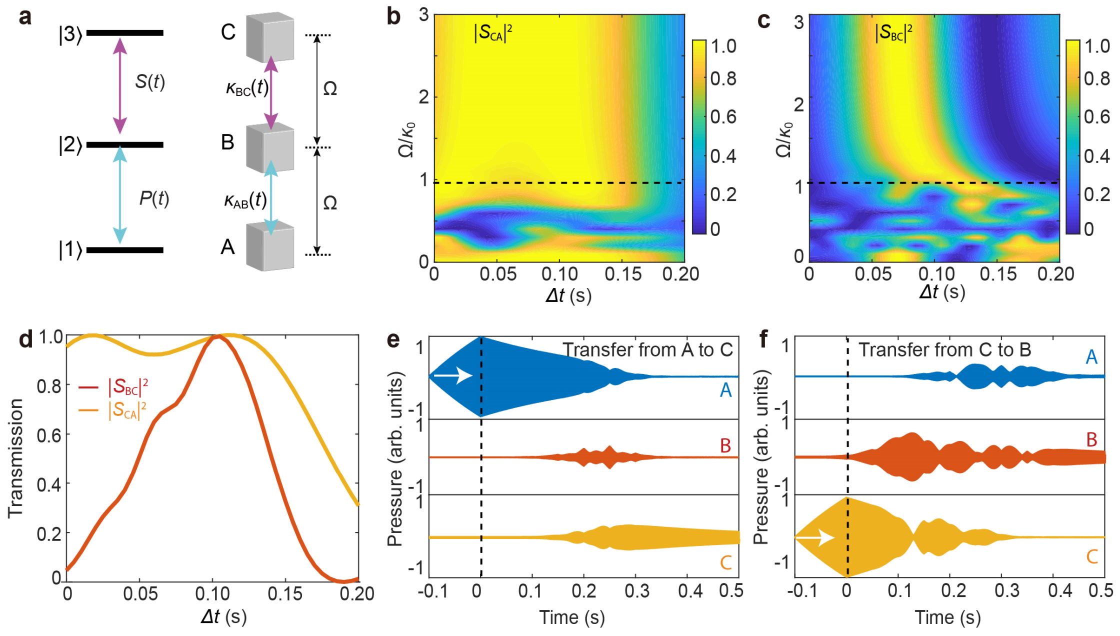

Fig. S11. **a**, Three cavities with different eigenfrequencies form a ladder-type three-level system. The frequency of **B** lies between **A** and **C** with detuning $\Omega_{AB} = \Omega_{BC} = \Omega$. The two dynamic couplings are $\kappa_{AB}(t) = \kappa_0 \mathrm{sign}[\cos(2\pi\Omega t)]e^{-(t-t_0-\Delta t)^2/\sigma^2}$ and $\kappa_{BC}(t) = \kappa_0 \mathrm{sign}[\cos(2\pi\Omega t)]e^{-(t-t_0)^2/\sigma^2}$. Other parameters are $\kappa_0 = 10$ Hz, $t_0 = 0.185$ s and $\sigma = 0.1$ s. **b-c**, Simulated transfer efficiencies $|S_{CA}|^2$ (**b**) and $|S_{BC}|^2$ (**c**) as a function of Δt and Ω. **d**, Simulated transfer efficiencies $|S_{CA}|^2$ and $|S_{BC}|^2$ with $\Omega = \kappa_0$, which is shown as the dashed lines in **b-c**. **e-f**, Simulated forward transfer from cavity **A** to **C** (**e**) and backward transfer from cavity **C** to **B** (**f**) with $\Delta t = 0.1$ s and $\Omega = \kappa_0$.

Firstly, we consider the ladder-type three-level system shown in Fig. S11**a**. We have $f_B = f_A + \Omega$ and $f_C = f_A + 2\Omega$, where Ω is the detuning between adjacent cavities. The other parameters are given in the caption of the figure. We simulate the transfer efficiencies $|S_{CA}|^2$ and $|S_{BC}|^2$ as a function of Δt and Ω. As shown in Figs. S11**b-**

c, as long as Ω is larger than κ_0, we achieve the STIRAP-type transfer, *i.e.*, robust energy transfer from cavity **A** to **C** (see Fig. S11**b**) or unitary transfer from cavity **C** to **B** (see Fig. S11**c**). Specifically, we show the transfer for $\Omega = \kappa_0$ in Fig. S11**d**. The non-reciprocity of the transfers is documented by the results shown in Figs. S11**e-f**.

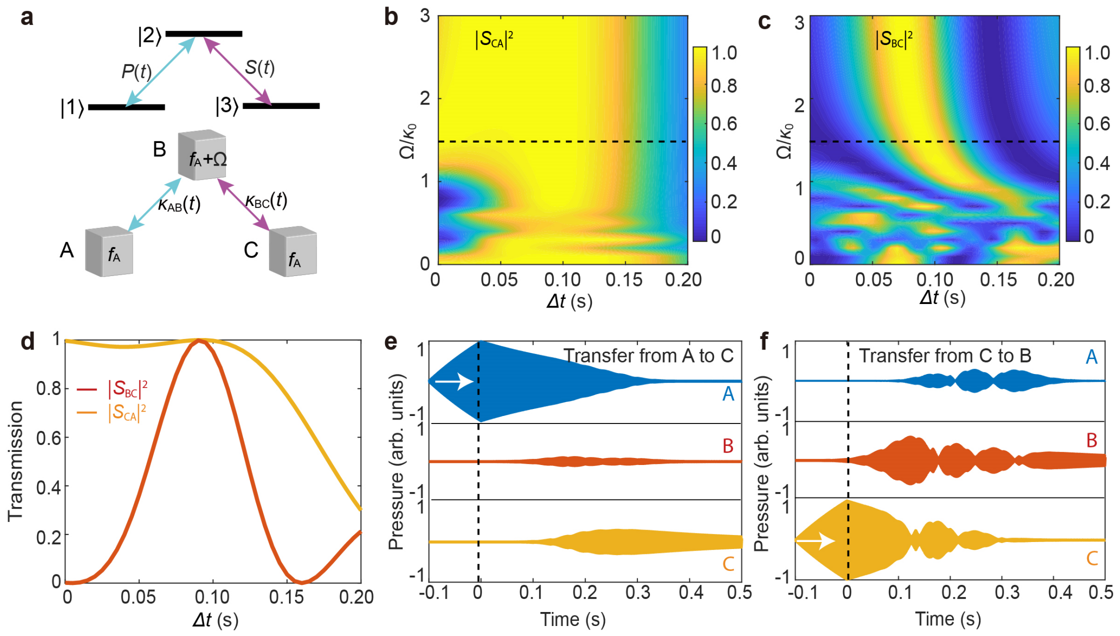

Fig. S12. **a**, Three cavities with different eigenfrequencies forming a lambda-type three-level system that **B**'s frequency lies above **A** and **C**. Other parameters are the same as Fig. S11. **b-c**, Simulated transfer efficiencies $|S_{CA}|^2$ (**b**) and $|S_{BC}|^2$ (**c**) as a function of Δt and Ω. **d**, Simulated transfer efficiencies $|S_{CA}|^2$ and $|S_{BC}|^2$ with $\Omega = 1.5\kappa_0$, which is denoted as the dashed lines in **b-c**. **e-f**, Simulated forward transfer from cavity **A** to **C** (**e**) and backward transfer from cavity **C** to **B** (**f**) with $\Delta t = 0.09$ s and $\Omega = 1.5\kappa_0$.

Secondly, we study the lambda-type three-level system in Fig. S12**a**, where we have $f_A = f_C$ and $f_B = f_A + \Omega$. By simulation, we confirm that we can get a robust and non-reciprocal transfer as long as the coupling strength is slightly smaller than the detuning. In Figs. S12**b-c**, we plot the simulated transfer efficiencies $|S_{CA}|^2$ and $|S_{BC}|^2$ as a function of Δt and Ω. By setting $\Omega = 1.5\kappa_0$, we plot the transmissions in Figs. S12**d**, which are quite similar to the results in Figs. 3-4 of the main text. The wave transfers with $\Delta t = 0.09$ s and $\Omega = 1.5\kappa_0$ are shown in Figs. S12**e-f**.

Finally, we conclude that we can realize robust acoustic transfer with the TQPM method if the detuning is larger than the coupling strength. Such transfer assisted by the STIRAP method shows more advantage in terms of robustness compared with methods

reported in Ref. [5].

References

1. B. T. Torosov, G. Della Valle, S. Longhi, Non-Hermitian shortcut to stimulated Raman adiabatic passage. *Phys. Rev. A* **89**, 063412 (2014).
2. Y.-X. Shen *et al.*, One-Way Localized Adiabatic Passage in an Acoustic System. *Phys. Rev. Lett.* **122**, 094501 (2019).
3. U. Gaubatz, P. Rudecki, S. Schiemann, K. Bergmann, Population transfer between molecular vibrational levels by stimulated Raman scattering with partially overlapping laser fields. A new concept and experimental results. *J. Chem. Phys.* **92**, 5363-5376 (1990).
4. K. Bergmann *et al.*, Roadmap on STIRAP applications. *J. Phys. B* **52**, 202001 (2019).
5. Y. Mazor, M. Cotrufo, A. Alù, Unitary Excitation Transfer between Coupled Cavities Using Temporal Switching. *Phys. Rev. Lett.* **127**, 013902 (2021).
